# Is simulation training effective in increasing podiatrists’ knowledge and confidence in foot ulcer management? A pilot study

**DOI:** 10.1186/1757-1146-4-S1-O26

**Published:** 2011-05-20

**Authors:** Peter A Lazzarini, Elizabeth L Mackenroth, Patricia M Régo, Frances M Boyle, Scott Jen, Ewan M Kinnear, Maarten C Kamp

**Affiliations:** 1 Allied Health Research Collaborative, Metro North Health Service District, Queensland Health, Brisbane, Australia; 2 Department of Podiatry, Metro North Health Service District, Queensland Health, Brisbane, Australia; 3 School of Public Health, Queensland University of Technology, Brisbane, Australia; 4 School of Medicine, The University of Queensland, Brisbane, Australia; 5 Clinical Skills Development Service, Centre for Healthcare Improvement, Queensland Health, Brisbane, Australia; 6 School of Population Health, The University of Queensland, Brisbane, Australia; 7 Department of Podiatry, Darling Downs-West Moreton Health Service District, Queensland Health, Ipswich, Australia; 8 Department of Endocrinology, Metro North Health Service District, Queensland Health, Brisbane, Australia

## Background

Diabetic foot ulcers are commonly acknowledged as the most frequent reason for admission into hospital for diabetes-related complications. Clinical training is known to have a beneficial impact on diabetic foot ulcer outcomes. Simulation clinical training has rarely been used in the management of diabetic feet or chronic wounds. The few simulation courses in this area have focused solely on training for a single technical skill. This pilot study aimed to investigate the effect of a mixed modality simulation training program on podiatry participants’ clinical knowledge, confidence and satisfaction in the management of foot ulcers.

## Methods

Sixteen podiatrists participated in a two-day Foot Ulcer Simulation Training (FUST) course. It included web-based interactive learning, low-fidelity part-tasks and high-fidelity full clinical scenarios. Primary outcome measures of evaluation of the course included participants’ pre- and post completion of confidence and knowledge surveys. Participants’ satisfaction and the relevance and fidelity of a range of course elements were also investigated.

## Results

A significant improvement in clinical confidence was observed following completion of FUST (mean scores 3.10 cf. 4.40, p < 0.05). The lack of a significant change in pre- and post knowledge scores reflected participants’ mandatory pre-course completion of web-based components. Satisfaction, relevance and fidelity of all course elements were rated highly by participants.

## Conclusions

This pilot study demonstrates the successful use of simulation in the training of foot ulcer management. The approach has the potential to revolutionise training in wound care and to improve patient outcomes.

